# A Fiber Bragg Grating-Based Measurement Method for Outer-Ring Fault Detection in Rolling Bearings

**DOI:** 10.3390/s26154972

**Published:** 2026-08-05

**Authors:** Hongli Li, Gang Xu, Xin Gui, Zhengying Li

**Affiliations:** 1 School of Information Engineering, Wuhan University of Technology, Wuhan 430070, China; 277851@whut.edu.cn; 2 School of Mechanical Engineering, Hubei Engineering University, Xiaogan 432100, China; 3 National Engineering Research Center of Optical Fiber Sensing Technology and Networks, Wuhan University of Technology, Wuhan 430070, China; guixin@whut.edu.cn

**Keywords:** Fiber Bragg Grating, rolling bearing, dynamic strain measurement, outer-ring fault, local mean decomposition, maximum correlated kurtosis deconvolution, minimum entropy deconvolution

## Abstract

**Highlights:**

**What are the main findings?**

- An embedded fiber Bragg grating (FBG) sensor enables near-source dynamic strain measurement of the bearing outer ring for outer-ring fault detection.
- The combined LMD-MCKD signal processing method effectively enhances periodic impulsive components, making the outer-ring fault characteristic frequency and its harmonics more distinguishable.

**What are the implications of the main findings?**

- The proposed method provides a feasible fiber-optic sensing approach for rolling bearing condition monitoring, especially for weak fault detection where conventional external vibration measurements may be affected by signal transmission attenuation and noise.
- The near-source FBG-based measurement strategy offers a potential technical route for developing smart bearings and health monitoring systems for rotating machinery operating under complex conditions

**Abstract:**

A fiber Bragg grating (FBG)-based measurement method is proposed for outer-ring fault detection in rolling bearings. To acquire fault-related responses in a near-source manner, a circumferential groove was machined on the bearing outer ring, and an FBG sensor was embedded and bonded to directly measure the dynamic strain of the outer ring. The acquired strain signal was first decomposed using local mean decomposition (LMD), and the product function components were selected for reconstruction according to a correlation coefficient–kurtosis weighted criterion. Maximum correlated kurtosis deconvolution (MCKD) was then applied to enhance the weak periodic impulsive components associated with the outer-ring fault. Experiments were conducted on an NJ205EM cylindrical roller bearing with an artificial outer-ring defect at a rotational speed of 600 r/min and a sampling frequency of 8 kHz. The results show that the raw FBG signal contains the outer-ring fault characteristic frequency, but the fault-related component is weak and easily affected by surrounding spectral components. After LMD-MCKD processing, the fault characteristic frequency and its harmonics become more distinguishable in the envelope spectrum. A comparison with the conventional minimum entropy deconvolution (MED) method further confirms the effectiveness of the proposed method. The fault feature amplitude ratio and the corresponding local signal-to-noise ratio increase from 12.257 and 21.767 dB for MED to 16.985 and 24.555 dB for the proposed LMD-MCKD method, respectively. These results demonstrate that the proposed FBG-based near-source strain measurement combined with LMD-MCKD processing provides an effective approach for weak outer-ring fault detection in rolling bearings.

## 1. Introduction

Rolling bearings are indispensable components in rotating machinery and are widely used in aerospace systems, high-speed railways, wind turbines, precision machine tools, and other industrial equipment. Since they often operate under complex environments and varying working conditions, their health status directly affects the safety, reliability, and service life of mechanical equipment [1,2,3,4]. When localized defects occur in the inner ring, outer ring, rolling elements, or cage, periodic impacts are generated as the rolling elements pass through the defective region. If such early-stage faults are not detected in time, they may gradually develop into severe failures, leading to unexpected shutdowns or even safety accidents. Therefore, accurate detection of weak bearing faults is of great significance for condition monitoring, predictive maintenance, and the intelligent operation of rotating machinery [5].

At present, bearing fault detection is mainly performed using vibration-based diagnosis with externally mounted accelerators, owing to its convenient installation and well-established theoretical basis [6,7]. The characteristic frequencies of bearing faults, calculated from the bearing geometry, rotational speed, and contact angle, provide an important theoretical basis for identifying fault types from the measured spectrum or envelope spectrum. However, in practical applications, early bearing fault signals are usually weak, transient, and intermittent. During transmission, useful fault information can be attenuated or distorted by mechanical interfaces and is easily contaminated by background vibration, structural resonance, and noise from other sources. As a result, reliable extraction of weak fault features remains a challenging issue in bearing fault diagnosis [8].

To improve the detectability of weak fault features, various signal processing methods have been developed. Huang et al. proposed empirical mode decomposition (EMD), which decomposes nonlinear and non-stationary signals into a series of intrinsic mode functions according to their local extrema [9,10]. This method has been widely applied in rotating machinery fault diagnosis. Nevertheless, EMD may suffer from mode mixing, end effects, and limited physical interpretability of the decomposed components. To overcome these limitations, several adaptive decomposition methods have been introduced. Variational mode decomposition (VMD) provides an effective decomposition framework with good noise robustness, but its performance depends strongly on the preset number of modes and the penalty factor [11]. Local mean decomposition (LMD), another adaptive time-frequency analysis method, decomposes a signal into several product function components with physically meaningful instantaneous amplitude and instantaneous frequency [12]. Compared with EMD, LMD is more effective in preserving the local amplitude-frequency modulation characteristics of non-stationary signals, which makes it suitable for analyzing weak periodic impact signals in rotating machinery. In addition, impulse-enhancement methods such as minimum entropy deconvolution (MED), maximum correlated kurtosis deconvolution (MCKD), and spectral kurtosis have been widely used to enhance transient fault impulses [13,14,15]. Among them, MCKD is particularly suitable for extracting repeated impulses because it enhances periodic impact components according to the expected fault period.

In recent years, with the development of machine learning, deep learning, and multi-modal information fusion, bearing fault diagnosis has gradually evolved toward intelligent, interpretable, and physics-informed approaches [16,17,18]. However, the effectiveness of these methods still depends heavily on the quality of the acquired signals. When early fault responses are attenuated or contaminated during transmission, subsequent feature extraction and diagnosis may become unreliable. Therefore, in addition to advanced signal processing and intelligent algorithms, improving the sensing strategy and acquiring fault-related responses in a near-source manner are also essential for weak fault detection.

Fiber Bragg grating (FBG) sensing provides a promising technical route for near-source measurement in harsh or complex mechanical environments. An FBG sensor reflects a narrowband optical signal, and its Bragg wavelength shifts with changes in strain and temperature. Compared with conventional electrical sensors, FBG sensors offer several advantages, including small size, light weight, immunity to electromagnetic interference, corrosion resistance, multiplexing capability, and flexible embedding [19]. These characteristics make FBG sensors suitable for structural health monitoring, dynamic strain measurement, and the development of smart mechanical components. In bearing monitoring, some studies have attempted to use FBG sensors to measure bearing temperature, load, deformation, or vibration response [20,21]. However, most existing studies mainly focus on sensing feasibility or general vibration monitoring, while the combination of embedded FBG-based near-source strain measurement and weak fault feature enhancement has not been sufficiently investigated.

For bearing outer-ring faults, the defect position is fixed relative to the bearing housing, and repeated contact between the rolling elements and the defective region produces periodic strain fluctuations in the outer ring. Therefore, direct measurement of the dynamic strain response of the outer ring is theoretically suitable for detecting outer-ring defects. In this study, a circumferential groove is machined on the outer surface of the bearing outer ring, and an FBG sensor is embedded and bonded in the groove to capture the local dynamic strain response of the outer ring. This near-source sensing strategy is expected to reduce the signal attenuation and interference commonly encountered in conventional external vibration measurement. Meanwhile, the theoretical outer-ring fault characteristic frequency is calculated according to the bearing geometry and rotational speed, and the fault is identified by observing whether this frequency and its harmonics are enhanced in the envelope spectrum after signal processing.

Based on the above considerations, this study proposes an FBG-based measurement method for outer-ring fault detection in rolling bearings. By embedding an FBG sensor into a circumferential groove machined on the bearing outer ring, the dynamic strain response can be measured directly in a near-source manner. The acquired strain signal is then decomposed by LMD, and the product function components are selected for reconstruction using a correlation coefficient–kurtosis weighted criterion. Subsequently, MCKD is applied to the reconstructed signal to further enhance the periodic impulsive features associated with the outer-ring fault. Experiments are conducted on a cylindrical roller bearing with an artificial outer-ring defect to evaluate the proposed method. The results demonstrate that the proposed approach improves the distinguishability of fault-related features and provides a feasible measurement route for rolling bearing condition monitoring.

The main contributions of this work are summarized as follows:

(1) An embedded FBG-based near-source strain measurement scheme is proposed for bearing outer-ring fault detection, which provides a potential sensing route for smart bearings and rotating machinery condition monitoring.
(2) A combined LMD-MCKD signal processing method is introduced to enhance weak periodic impulses in FBG strain signals. To the best of the authors’ knowledge, this is the first attempt to apply LMD-MCKD to bearing outer-ring strain signals measured by embedded FBG sensing.
(3) A correlation coefficient–kurtosis weighted criterion is used for product function component selection, so that both signal similarity and impulsive characteristics are considered during signal reconstruction.
(4) The outer-ring fault is identified based on the theoretical fault characteristic frequency and its harmonics in the envelope spectrum, providing a clear physical basis for fault judgment.

## 2. FBG Measurement Method for Bearing Fault

### 2.1. Test Rig Design

**Figure 1:**
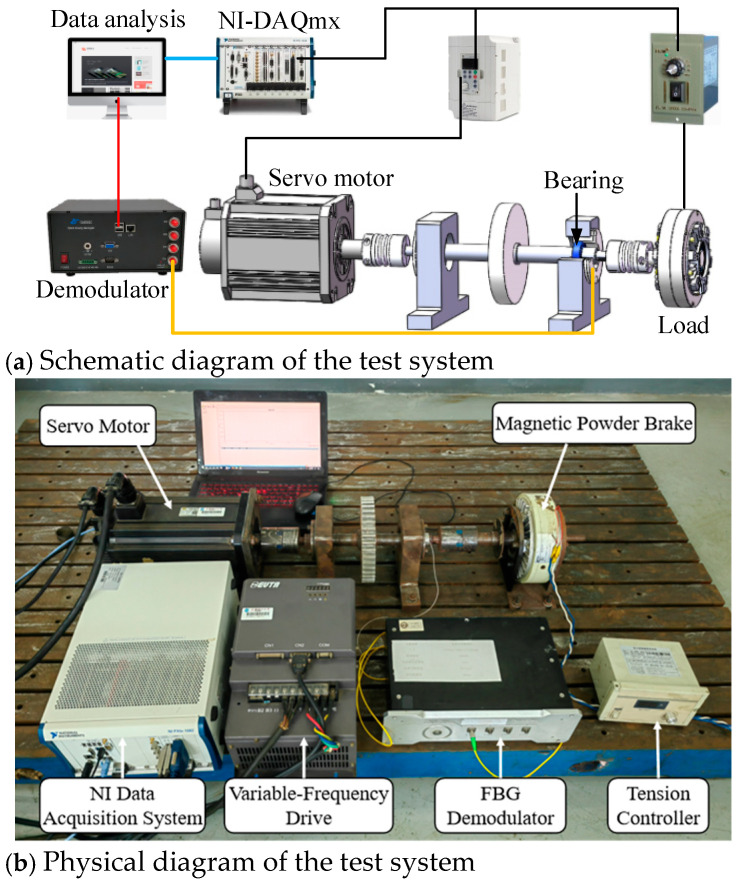
Bearing fault test system.

The bearing fault test rig used in this study was developed in-house to simulate rolling bearing operation under controlled rotational speed and load conditions. As shown in Figure 1, the system consists of a 7.5 kW servo motor with a maximum output torque of 48 N m, two couplings, a test shaft supported by the test bearing, two bearing housings, and a magnetic powder brake with a maximum load of 50 N m. The rotational speed was controlled by the servo drive, and the braking torque was adjusted by the magnetic powder brake. The motor and brake control signals were acquired through an NI-DAQmx-based data acquisition system. The FBG signal was demodulated using a self-developed optical fiber demodulator, and the sampling frequency was set to 8 kHz. All signal processing was performed using a unified MATLAB R2021b-based program to ensure consistency of the results.

### 2.2. Bearing and Its Defect Design

**Figure 2:**
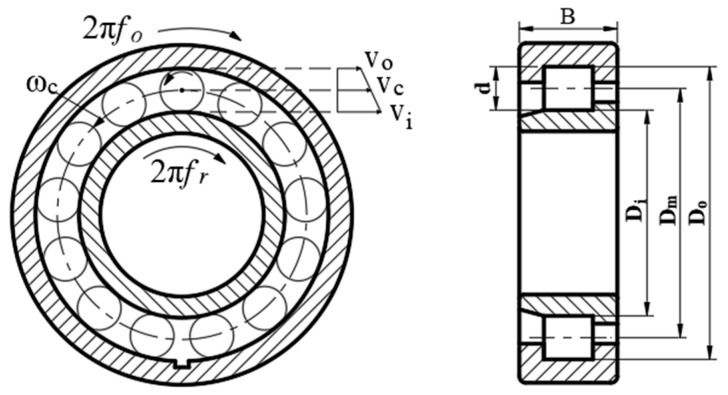
Bearing structure and dimensions.

The test bearing was an NJ205EM cylindrical roller bearing with thirteen rollers. Its structure and main dimensions are shown in Figure 2. The roller diameter *d* is 6.6 mm, the diameter *Di* of the inner ring raceway is 31.5 mm, the diameter *Do* of the outer ring raceway is 44.8 mm, the contact angle *α* is 0°, and the bearing width *B* is 12 mm. During operation, the inner ring is fitted to the rotating shaft and rotates with the shaft, while the outer ring is fixed in the bearing housing. The cage maintains uniform roller spacing and rotates with the rolling elements.

According to the bearing geometry, the pitch diameter *D_m_* of the bearing is equal to:

(1) $$D_{m}\;=\;\frac{D_{i\;}\;+\;D_{o}}{2}$$

The rotational linear velocity of the inner and outer raceways can be expressed as:

(2) $$V_{i}\;=\;2\pi f_{i}\frac{D_{i}}{2}$$

(3) $$V_{o}=2\pi f_{o}\frac{D_{o}}{2}$$

where *f_i_* is the rotational frequency of the inner raceway, and *f_o_* is the rotational frequency of the outer raceway.

If the rollers roll ideally on the raceway, the rotational speed *V_c_* of the cage is the average of the rotational speeds of the inner and outer rings.

(4) $$V_{c}=\frac{V_{i\;}+V_{o}}{2}=\frac{2\pi f_{i}\left(D_{m}-d\cos\alpha \right)}{4}+\frac{2\pi f_{o}\left(D_{m}+d\cos\alpha \right)}{4}$$

In this paper, the outer ring of the bearing is fixed. After simplifying the above formula, the characteristic frequency of the cage *f_c_* can be expressed as [22,23]:

(5) $$f_{c}\;=\;\frac{1}{2}\left(1-\frac{d}{D_{m}}\cos\alpha \right)f_{i}$$

If a bearing has Z rollers, then when the cage rolls around the inner ring for one full revolution, each roller will pass through the fixed point on the inner ring Z times. The inner-ring fault characteristic frequency *f_bpfi_* can be expressed as:

(6) $$f_{\mathrm{bpfi}}\;=\;\frac{1}{2}\left(1\;+\;\frac{d}{D_{m}}\cos\alpha \right)f_{i}Z$$

Similarly, the outer-ring fault characteristic frequency *f_bpfo_* can be expressed as:

(7) $$f_{\mathrm{bpfo}}\;=\;\frac{1}{2}\left(1-\frac{d}{D_{m}}\cos\alpha \right)f_{i}Z$$

The rolling-element fault characteristic frequency *f_bsf_* can be expressed as:

(8) $$f_{\mathrm{bsf}}\;=\;\frac{1}{2}\left(1-{\left(\frac{d}{D_{m}}\cos\alpha \right)}^{2}\right)f_{i}\frac{D_{m}}{d}$$

Based on the bearing fault characteristic frequency Formulas (5)–(8) and the structural parameters of the NJ205EM bearing, the calculated defect frequencies of the NJ205EM bearing are listed in Table 1.

**Figure 3:**
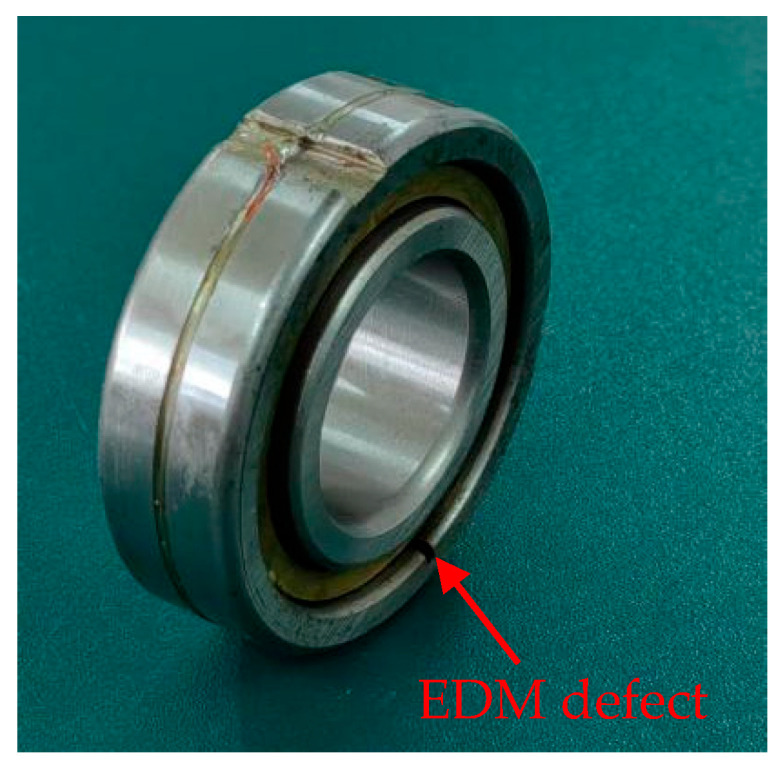
Physical picture of defective bearing.

To evaluate the feasibility of the proposed FBG-based sensing strategy under controlled and repeatable conditions, an artificial outer-ring defect was introduced into the test bearing by electrical discharge machining (EDM), as shown in Figure 3. The defect was located on the inner circumferential surface of the outer ring, within the primary load-bearing contact region between the rollers and the outer ring. Its depth and width were both approximately 1 mm. It should be noted that the EDM defect is a simplified representation of a localized outer-ring defect rather than a complete reproduction of naturally developed pitting or spalling. This design was adopted to provide a repeatable excitation source for preliminary validation of the near-source FBG measurement and signal processing method. Further validation using naturally degraded bearings will be carried out in future work.

### 2.3. Layout and Design of FBG Unit

A fiber Bragg grating (FBG) is formed by periodic modulation of the refractive index in the fiber core. When broadband light propagates along the fiber, the FBG reflects a narrowband component whose center wavelength, known as the Bragg wavelength, is determined by the effective refractive index and the grating period [24,25]. The Bragg wavelength can be expressed as:

(9) $$\lambda \;=\;2n_{\mathrm{eff}}\Lambda$$

where *λ* is the Bragg wavelength, *n_eff_* is the effective refractive index of the fiber core, and *Λ* is the grating period.

The Bragg wavelength shift is affected by both axial strain and temperature variation. In this case, the Bragg wavelength dependency on the strain and temperature could be described by:

(10) $$\frac{\Delta \lambda }{\lambda }\;=\left(\alpha_{f}+\xi \right)\Delta T+\left(1-P_{e}\right)\Delta \varepsilon$$

where *α_f_* is the thermal expansion coefficient of the fiber, ξ is the thermo-optic coefficient, Pe (approximately 0.22 at room temperature) is the effective photo-elastic coefficient, ∆ε is the axial strain, and ∆T is the temperature variation.

For dynamic bearing fault detection, the fault-related strain response is mainly reflected in the high-frequency dynamic component, whereas temperature variation changes slowly during the short-duration test. Therefore, the dynamic component of the FBG signal was used for fault feature analysis, and the low-frequency trend was removed before envelope analysis. For long-term field monitoring, an additional temperature-compensation FBG or a differential sensing configuration should be used to further separate thermal and mechanical effects.

**Figure 4:**
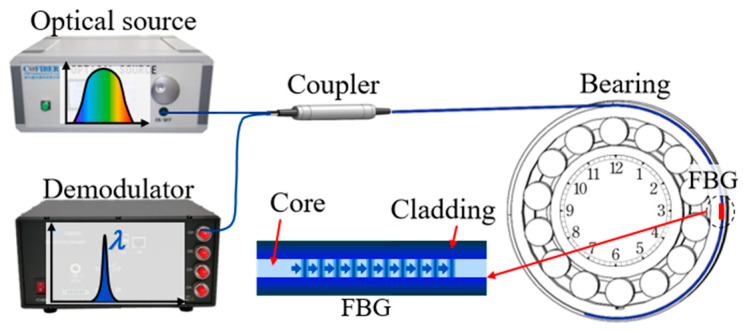
Bearing instrumentation with the FBG sensing system.

This work aims to evaluate the feasibility of using FBG sensing for bearing condition monitoring by directly measuring the dynamic strain of the bearing outer ring. Figure 4 shows the installation diagram of FBG on the bearing. The length of the FBG used in the experiment was approximately 5 mm. The sensor was arranged near the expected load-contact region to improve the sensitivity to the outer-ring defect-induced strain response.

To embed the FBG sensor, the test bearing was disassembled and a 0.5 mm × 0.5 mm circumferential groove was machined on the outer surface of the outer ring. The optical fiber containing the FBG was placed in the groove and bonded to the outer ring using strain adhesive. After installation, the groove was filled with adhesive to improve strain transfer continuity and reduce the local disturbance caused by the groove. Although the shallow groove was designed to minimize its influence on the bearing structure, the machining process may still locally alter the structural properties of the outer ring. Therefore, the present work focuses on the feasibility of near-source dynamic strain measurement and weak fault feature extraction, while fatigue life and load distribution effects will be investigated in future structural reliability studies. In addition, because a single FBG measures local strain within a limited sensing length, the present sensor arrangement is most sensitive to defects passing through the primary contact region; circumferential FBG arrays will be considered in future work to improve adaptability to defect locations.

## 3. Signal Processing Method and Experiment on Bearing Defect Identification

### 3.1. Signal Acquisition and Spectrum Analysis

**Figure 5:**
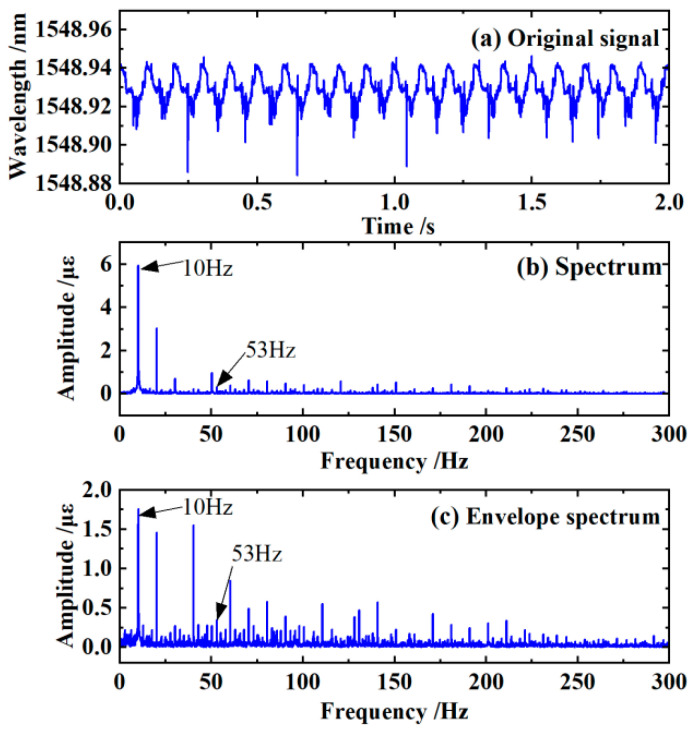
Original signal, spectrum and envelope spectrum of the bearing.

The outer-ring defect data acquired from the test system were analyzed in this section. The sampling frequency was 8 kHz, and the shaft speed was 600 r/min, corresponding to a rotational frequency of 10 Hz. According to Equation (7) and the geometric parameters of the NJ205EM bearing, the theoretical outer-ring fault characteristic frequency was calculated as approximately 53.6 Hz. Figure 5 presents the time-domain waveform, spectrum, and envelope spectrum of the raw FBG strain signal. The periodic impact components are not obvious in the time domain, and the fault characteristic frequency can be observed only weakly in the envelope spectrum. This indicates that direct identification from the raw signal is difficult and that further feature enhancement is necessary.

### 3.2. LMD-MCKD Analysis Method

Local mean decomposition (LMD) is an adaptive time-frequency analysis method that can decompose a non-stationary signal into several product function (PF) components while preserving local amplitude-frequency modulation characteristics [26,27]. Maximum correlated kurtosis deconvolution (MCKD) is a deconvolution method designed to enhance periodic impulses according to a specified fault period [28,29]. In this study, LMD is first used to separate fault-related PF components from the measured FBG strain signal, and MCKD is subsequently applied to the reconstructed signal to further enhance the periodic impacts caused by the outer-ring defect.

#### 3.2.1. LMD Signal Decomposition

The essence of LMD is to decompose a signal into envelope signals and pure frequency-modulated (FM) signals at different scales. The product of an envelope signal and a pure frequency-modulated signal is defined as a PF component, which has physically meaningful instantaneous amplitude and instantaneous frequency. The decomposition is repeated until all PF components are separated. The main steps are as follows:

(1) Calculate the local mean $m_{i}=\frac{n_{i}+n_{i+1}}{2}$ and local envelope value $a_{i}=\frac{\left|n_{i}-n_{i+1}\right|}{2}$ from adjacent extrema $n_{i}$ of the signal, and connect all local mean and local envelope value with a straight line. Then the moving average method is used for smoothing to obtain the local mean function $m_{11}\left(t\right)$ and local envelope function $a_{11}\left(t\right)$.
(2) Subtract the local mean function $m_{11}\left(t\right)$ from the original signal $x\left(t\right)$ and divide the result by the local envelope function $a_{11}\left(t\right)$ to obtain a demodulated signal $s_{11}\left(t\right)$, as expressed in Equation (11).
  (11) $$s_{11}\left(t\right)=\frac{\left(x\left(t\right)-m_{11}\left(t\right)\right)}{a_{11}\left(t\right)}$$
(3) The ideal demodulated signal is a pure FM signal, that is, it satisfies the local envelope function $a_{1n}\left(t\right)=1$. If the demodulated signal does not satisfy the condition, the above procedure is repeated until the local envelope approaches unity.
(4) Multiply all local envelope functions $a_{1i}\left(t\right)$ to obtain the envelope signal $a_{1}\left(t\right)$ of PF component, and then multiply it with pure FM signal $s_{1n}\left(t\right)$ to obtain the first PF component:
  (12) $${PF}_{1}\left(t\right)={a_{1}\left(t\right)s}_{1n}\left(t\right)=\left(\prod_{i\;=\;1}^{n}a_{1i}\left(t\right)\right)s_{1n}\left(t\right)$$

The first PF component contains the highest-frequency part of the given signal. It is a single component AM-FM signal whose instantaneous amplitude is envelope signal $a_{1}\left(t\right)$ and whose instantaneous frequency $f\left(t\right)=\frac{1}{2\pi }\frac{d\cos^{-1}\left(s_{1n}\left(t\right)\right)}{dt}$ can be obtained from the demodulated frequency-modulated signal.

(5) After the first PF component is separated from the original signal, the residual signal $u_{1}\left(t\right)$ is used as the new input and the decomposition is repeated until the residual becomes a monotonic function. Finally, the original signal is represented as the sum of k PF components and a residual term $u_{k}$, as shown in Equation (13).
  (13) $$x\left(t\right)=\sum_{p=1}^{k}{PF}_{p}\left(t\right)+u_{k}\left(t\right)$$

**Figure 6:**
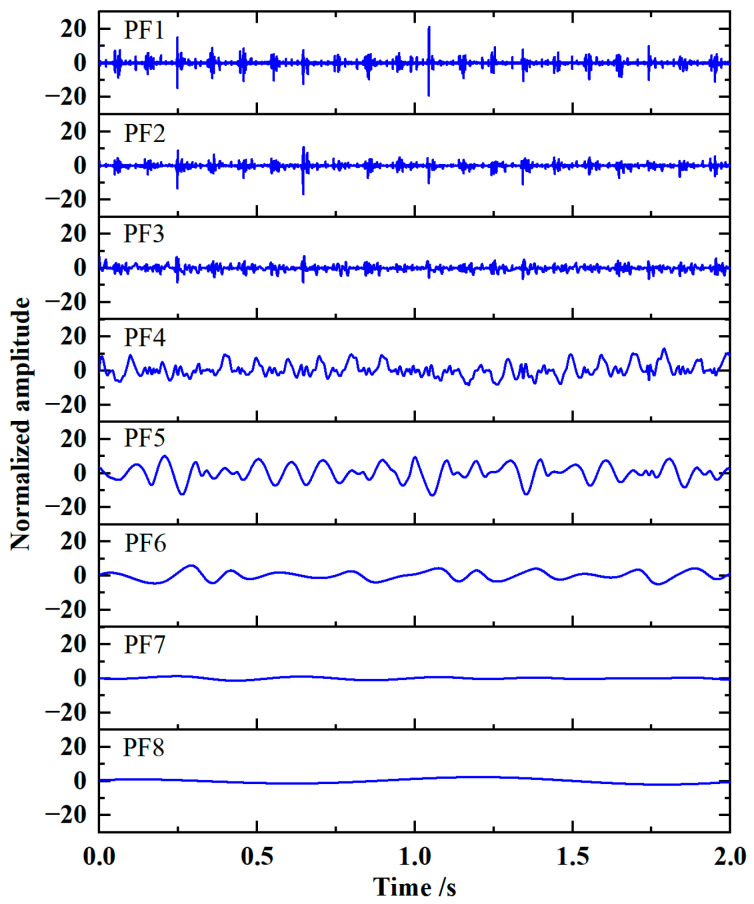
The PF components after LMD.

Figure 6 shows the PF components obtained from the raw FBG strain signal after LMD. Because some decomposed components may contain noise or unrelated oscillations, selecting appropriate PF components is essential for preserving fault-related information while suppressing irrelevant components.

#### 3.2.2. Signal Reconstruction Method

The FBG strain signal measured from a defective cylindrical roller bearing is nonlinear and non-stationary. Although LMD can adaptively decompose the signal into PF components, not all components are useful for fault diagnosis. Some PF components may be dominated by noise, structural vibration, or unrelated frequency components. Therefore, an effective component selection criterion is required before signal reconstruction to avoid losing useful fault information or introducing irrelevant components.

The correlation coefficient and kurtosis are commonly used for component selection. The correlation coefficient reflects the similarity between each PF component and the original signal, while kurtosis is sensitive to impulsive characteristics. However, using either indicator alone may be insufficient: the correlation coefficient can be affected by noise, and kurtosis may overemphasize isolated impulses while ignoring signal consistency. Therefore, this study combines the two indicators so that both similarity to the original signal and impulsiveness of the component are considered.

The expression of correlation coefficient *ρ* is:

(14) $$\rho =\frac{max\left[R_{{PF}_{i,x}}\left(\tau \right)\right]}{max\left[R_{x,x}\left(\tau \right)\right]}$$

Kurtosis index *Ku* is defined as:

(15) $$Ku=\frac{1}{N_{s}}\sum_{i\;=\;1}^{N_{s}}\frac{{\left(x_{i}\;-\;\mu \right)}^{4}}{\sigma^{4}}$$

where $R_{{PF}_{i,x}}\left(\tau \right)$ is the cross-correlation coefficient between each PF and the original signal, and $R_{x,x}\left(\tau \right)$ is the autocorrelation coefficient of the original signal, and σ and μ are the standard deviation and mean value of the original signal respectively. $\rho_{i}$ is the normalized correlation coefficient between the i-th PF component and the original signal, and ${Ku}_{i}$ is the raw kurtosis of the i-th PF component.

To avoid the influence of different numerical scales, the raw kurtosis index ${Ku}_{i}$ of all PF components are first normalized. The normalized kurtosis index $\tilde{{Ku}_{i}}$ is then multiplied by the normalized correlation coefficient to obtain the weighted index $\omega_{i}=\tilde{{Ku}_{i}}\times \rho_{i}$. PF components with weighted indices greater than the average value $\omega_{m}=\frac{1}{N_{PF}}\sum_{i=1}^{N_{PF}}\omega_{i}$ are selected for signal reconstruction. The raw kurtosis values ${Ku}_{i}$, normalized kurtosis index $\tilde{{Ku}_{i}}$ and correlation coefficients $\rho_{i}$ of the PF components are shown in Table 2.

**Figure 7:**
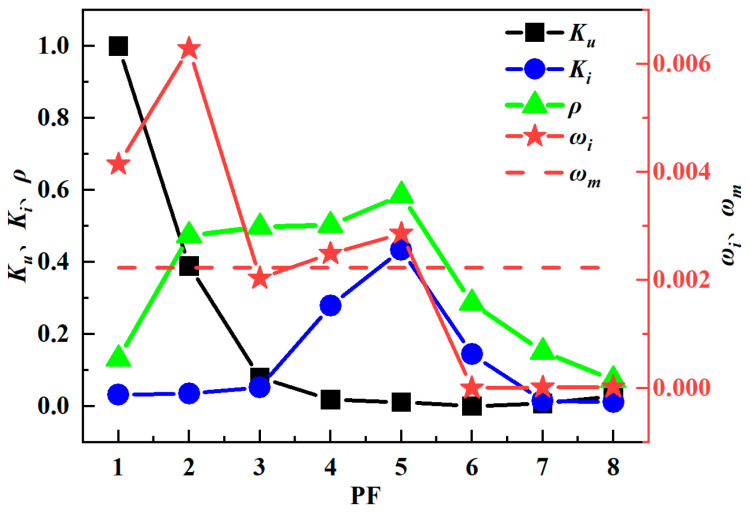
Correlation coefficients, kurtosis values, and weighted indices of PF components.

According to the above reconstruction criterion, the weighted index of each PF component was calculated, as shown in Figure 7. PF_1_, PF_2_, PF_4_, and PF_5_, whose weighted indices were greater than the average value, were selected to reconstruct the signal.

**Figure 8:**
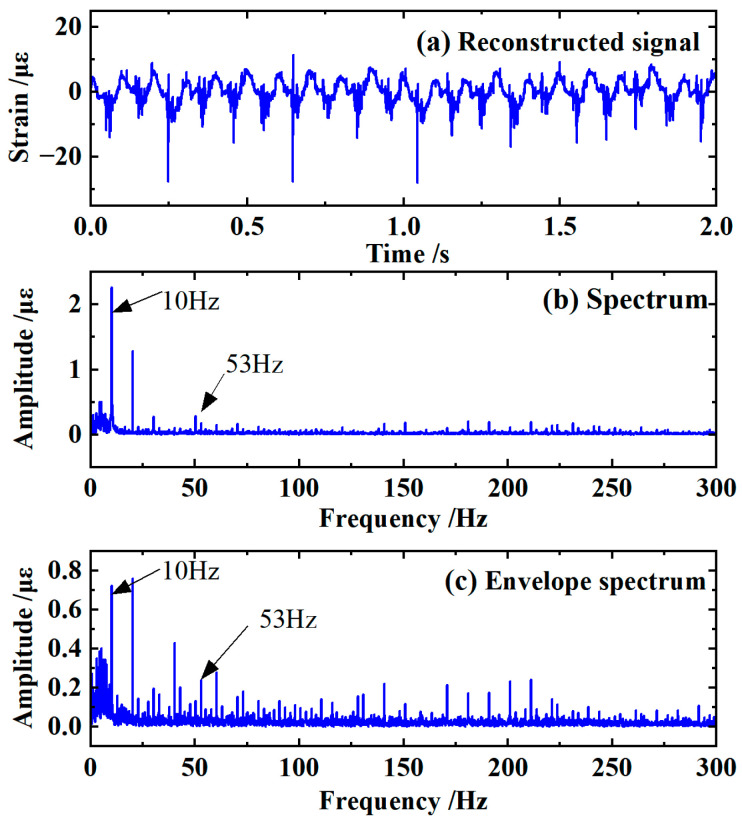
Time-domain waveform, spectrum, and envelope spectrum of the reconstructed signal.

The reconstructed signal and its spectrum and envelope spectrum are shown in Figure 8. As shown in Figure 8, after LMD-based reconstruction, the fault-related components are enhanced to some extent in the spectrum and envelope spectrum. However, the periodic impulsive features are still not sufficiently clear in the time-domain waveform, and the fault characteristic component remains affected by other spectral components. Therefore, LMD reconstruction alone is insufficient for reliable weak fault feature extraction, and further impulse enhancement is required.

#### 3.2.3. Maximum Correlated Kurtosis Deconvolution, MCKD

Because the FBG measures the dynamic strain of the bearing outer ring, the strain response associated with shaft rotation and structural vibration may be stronger than the weak impact response caused by the outer-ring defect. MCKD enhances periodic impulses by maximizing the correlated kurtosis of the filtered signal. It is therefore suitable for extracting repeated impact components whose period is consistent with the theoretical fault period.

In MCKD, a finite impulse response filter is iteratively optimized to recover the periodic impact sequence from the measured signal. The objective function of the MCKD algorithm can be expressed as follows:

(16) $${MCKD}_{M}\left(T\right)=\underset{f}{\max}{CK}_{M}\left(T\right)=\underset{f}{\max}\frac{\sum_{n\;=\;1}^{N}{\left(\prod_{m\;=\;0}^{M}y_{n\;-\;mT}\right)}^{2}}{{\left(\sum_{n\;=\;1}^{N}y_{n}^{2}\right)}^{M\;+\;1}}$$

where $y_{n}$ is the reconstructed signal; *N* is the sampling length; *M* is the number of periodic offsets; *T* is the period of the pulse signal; $f={\left[f_{1}f_{2}\ldots f_{L}\right]}^{T}$ is the filter sequence; *L* is the filter length; $T=f_{s}{\cdot T}_{s}$ is the deconvolution period; $f_{s}$ is the sampling rate; and $T_{s}$ is the fault period.

In this experiment, the outer-ring fault frequency was approximately 53.6 Hz, and the sampling frequency was 8 kHz. The physical fault period is denoted by $T_{s}=1/f_{bpfo}$, while the deconvolution period T is expressed in sampling points and calculated as $T=round\left(f_{s}T_{s}\right)=round\left(f_{s}/f_{bpfo}\right)=round\left(8000/53.6\right)=149samples$. The filter length L was set to 100 in this work. This value was selected to be shorter than one fault period so that the filter could capture the transient impulsive response caused by the outer-ring defect while avoiding the inclusion of adjacent fault impacts. A too short filter length may be insufficient to describe the impulse response, whereas an excessively long filter may introduce redundant components and reduce the stability of the deconvolution result. Based on the fault period calculated above and preliminary parameter trials, L = 100 provided a stable enhancement of the periodic impact components. The offset count M was set to 1, which means that the correlation kurtosis was calculated using adjacent fault impulses. Since the aim of this study is to enhance the fundamental periodic impulsive component associated with the outer-ring defect, M = 1 is sufficient for highlighting the periodic fault-related impacts. Larger values of M may increase the dependence on multiple consecutive impulses and are more sensitive to noise and fluctuation in weak fault signals.

**Figure 9:**
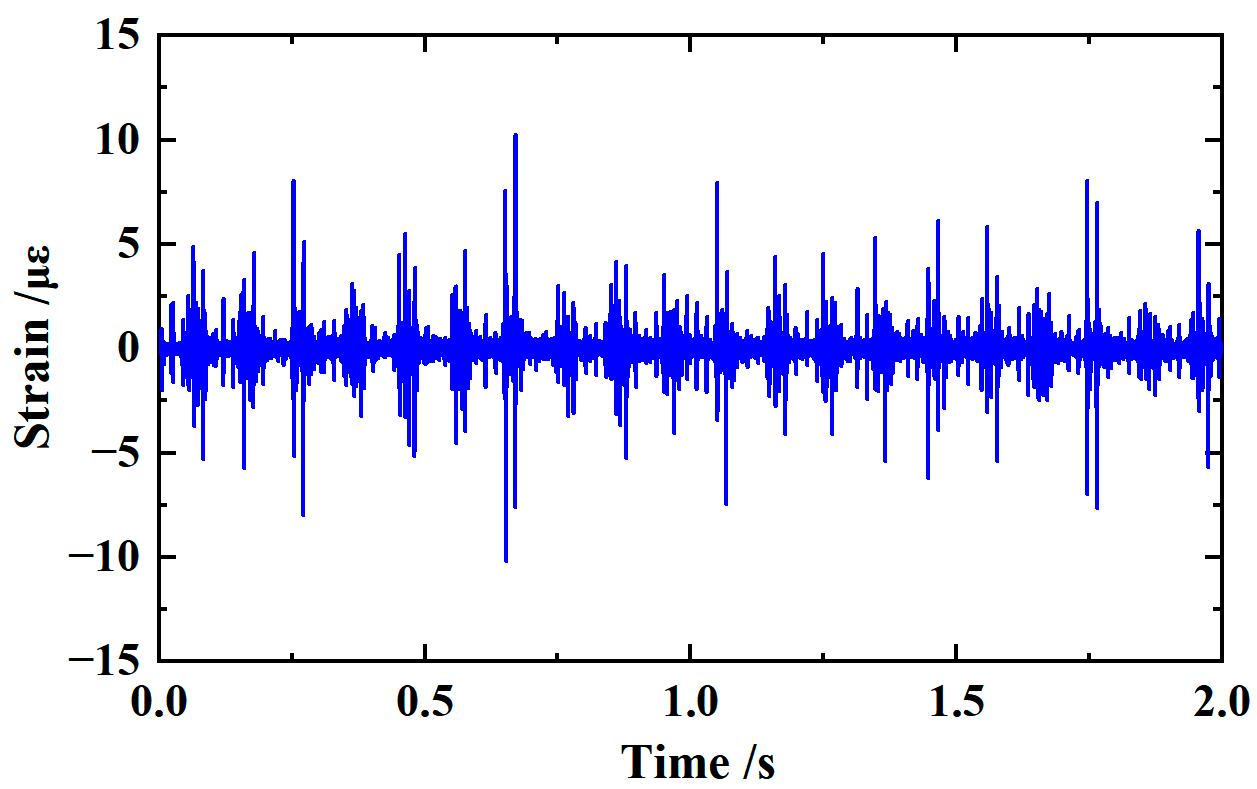
Time-domain waveform of the reconstructed signal after MCKD processing.

To further enhance the weak periodic impulsive components, MCKD was applied to the LMD-reconstructed signal. The time-domain waveform after MCKD processing is shown in Figure 9. Compared with the LMD-reconstructed signal in Figure 8, the periodic impulsive components become more evident, indicating that MCKD can effectively enhance the weak fault-related impacts contained in the reconstructed FBG strain signal.

**Figure 10:**
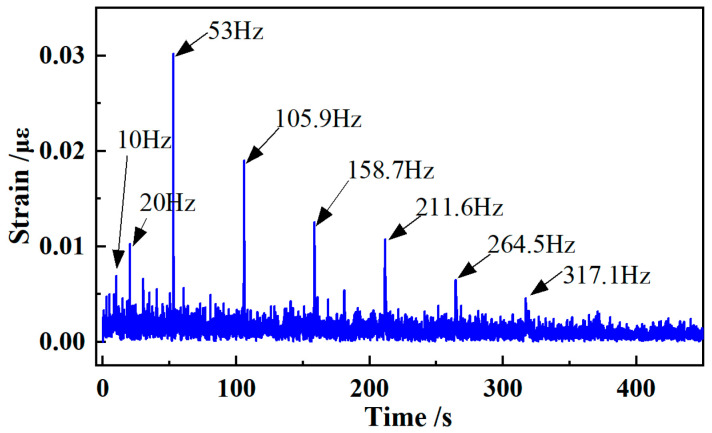
Envelope spectrum of the outer-ring fault signal after MCKD processing.

Figure 10 presents the corresponding envelope spectrum. A prominent peak appears at approximately 53.6 Hz, which agrees well with the theoretical outer-ring fault characteristic frequency calculated from the bearing geometry and rotational speed. Its harmonic components are also clearly visible, while no dominant peaks corresponding to the inner-ring, rolling-element, or cage fault characteristic frequencies are observed. Therefore, the bearing can be identified as having an outer-ring fault. This judgment is based on the consistency between the theoretical outer-ring fault frequency and the enhanced spectral components after LMD-MCKD processing.

### 3.3. Comparison with MED Method

To further verify the effectiveness of the proposed LMD-MCKD method, the conventional minimum entropy deconvolution (MED) method was introduced for comparison. MED is a commonly used deconvolution technique for enhancing impulsive components in rotating machinery fault diagnosis [30]. Therefore, it was selected as a baseline method in this study. For consistency, the same FBG strain signal was processed using MED, and the results were compared with those obtained from the raw signal, the LMD-reconstructed signal, and the proposed LMD-MCKD method under identical operating conditions.

**Figure 11:**
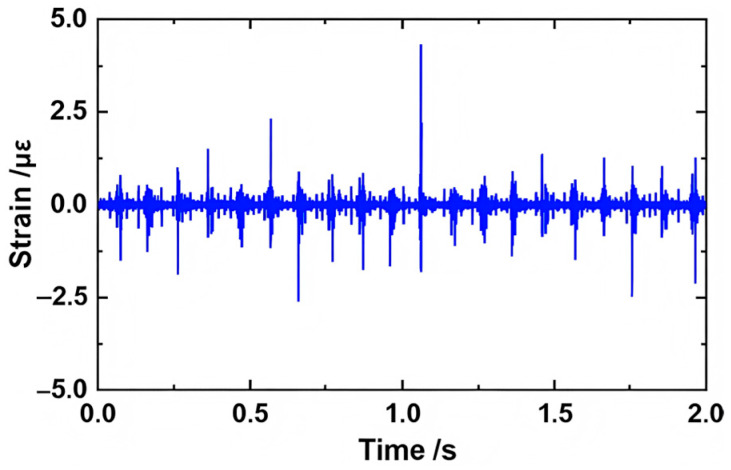
Time-domain waveform after MED processing.

**Figure 12:**
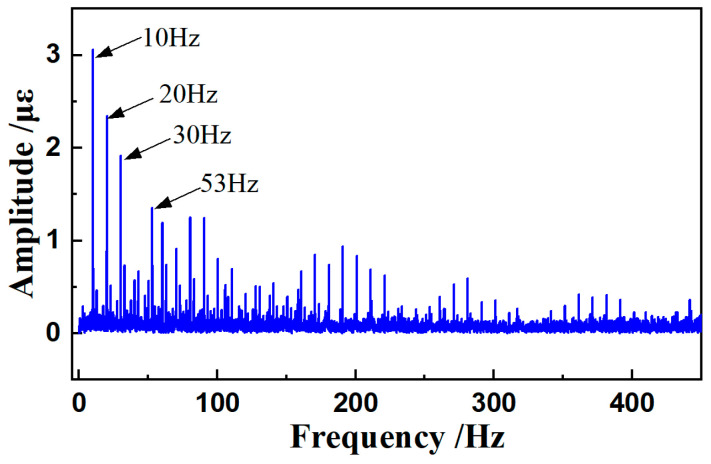
Envelope spectrum after MED processing.

Figure 11 shows the time-domain waveform of the signal after MED processing. Compared with the raw FBG signal, the impulsive components are enhanced to a certain extent, indicating that MED is capable of improving the visibility of transient fault-related responses. Figure 12 presents the corresponding envelope spectrum after MED processing. The outer-ring fault characteristic frequency can be identified, and the spectral components associated with the fault become more evident than those in the raw signal.

To quantitatively evaluate the fault feature enhancement performance, three indicators were used: the amplitude of the outer-ring fault characteristic frequency *A_bpfo_*, the fault feature amplitude ratio *R_f_*, and the corresponding signal-to-noise ratio *SNR_f_*. Here, *R_f_* is defined as the ratio between the amplitude at the theoretical outer-ring fault frequency and the average amplitude of the neighboring non-fault frequency components. *SNR_f_* is calculated from R_f_ in decibels. A larger *R_f_* or *SNR_f_* indicates that the fault characteristic component is more distinguishable from the surrounding background components.

The quantitative comparison is listed in Table 3. For the raw signal, the fault feature amplitude ratio is 6.421 and the corresponding *SNR_f_* is 16.152 dB, indicating that the outer-ring fault component exists but is not sufficiently prominent. After LMD reconstruction, *R_f_* and *SNR_f_* increase slightly to 7.184 and 17.127 dB, respectively, suggesting that LMD can suppress part of the irrelevant components but the enhancement effect remains limited. MED further improves the fault feature visibility, with *R_f_* and *SNR_f_* increasing to 12.257 and 21.767 dB, respectively. In comparison, the proposed LMD-MCKD method achieves the highest *R_f_* of 16.985 and *SNR_f_* of 24.555 dB among all methods.

It should be noted that the absolute amplitude *A_bpfo_* is affected by the filtering and reconstruction process and therefore should not be used alone to evaluate the enhancement performance. Instead, *R_f_* and *SNR_f_* provide a more reliable indication of the relative prominence of the fault characteristic frequency against the local spectral background. Although MED increases the absolute amplitude of the fault-related component, the proposed LMD-MCKD method provides a higher fault feature amplitude ratio and a higher local SNR. This demonstrates that the proposed method is more effective in highlighting weak periodic impulsive features from the near-source FBG strain signal and in improving the distinguish ability of the outer-ring fault characteristic frequency in the envelope spectrum.

## 4. Conclusions

This study proposed an FBG-based near-source dynamic strain measurement method for outer-ring fault detection in rolling bearings. By embedding an FBG sensor into a circumferential groove machined on the bearing outer ring, the dynamic strain response of the outer ring was directly acquired, reducing the influence of signal transmission attenuation and interference that commonly affect conventional external vibration measurements. The experimental results show that the raw FBG signal already contains the outer-ring fault characteristic frequency, but the spectral feature is weak and has limited diagnostic clarity.

To enhance the weak fault-related components, an LMD-MCKD signal processing strategy was developed. LMD was used to decompose the measured strain signal, and a correlation coefficient–kurtosis weighted criterion was adopted to select effective PF components for signal reconstruction. MCKD was then introduced to further enhance periodic impulsive components. After LMD-MCKD processing, the outer-ring fault characteristic frequency and its harmonics became more distinguishable in the envelope spectrum, indicating that the proposed method can effectively improve the visibility of weak fault features.

The comparison with MED further verifies the advantage of the proposed method. Although MED enhanced the impulsive components and increased the absolute amplitude of the fault-related component, the proposed LMD-MCKD method achieved a higher fault feature amplitude ratio and local signal-to-noise ratio. Specifically, *R_f_* increased from 12.257 for MED to 16.985 for the proposed method, while *SNR_f_* increased from 21.767 dB to 24.555 dB. This indicates that the proposed method provides better relative enhancement of the outer-ring fault characteristic component against the local spectral background.

Overall, these results demonstrate that the combination of FBG-based near-source strain measurement and LMD-MCKD processing is feasible and effective for weak outer-ring fault detection in rolling bearings. The method offers a useful technical route for fiber-optic sensing-based bearing condition monitoring. Future work will focus on validation under different rotational speeds, load conditions, fault severities, and naturally degraded bearing faults to further evaluate its robustness and engineering applicability.

## Figures and Tables

**Table 1:** NJ205EM bearing defect frequencies (multiple of running speed in Hz).

Fault Type	Defect Frequencies
Inner Ring	7.6347
Outer Ring	5.3653
Cage Train	2.7768
Rolling Element	5.5537

**Table 2:** ${Ku}_{i}$, $\tilde{{Ku}_{i}}$, $\rho_{i}$ of PF components.

PF	PF_1_	PF_2_	PF_3_	PF_4_	PF_5_	PF_6_	PF_7_	PF_8_
${\mathrm{Ku}}_{i}$	53.210	22.171	6.436	3.320	2.987	2.416	2.773	3.812
$\tilde{{\mathrm{Ku}}_{i}}$	1	0.389	0.079	0.018	0.011	0	0.007	0.027
$\rho_{i}$	0.131	0.474	0.497	0.502	0.585	0.286	0.151	0.072

**Table 3:** Quantitative comparison of fault feature enhancement performance using different methods.

Method	*A_bpfo_*	*R_f_*	*SNR_f_*/*dB*
Raw signal	0.349	6.421	16.152
LMD reconstructed signal	0.241	7.184	17.127
MED	1.36	12.257	21.767
Proposed LMD-MCKD	0.030	16.985	24.555

## Data Availability

Data are contained within the article.

## Abbreviations

**Abbreviation**	**Full term**	
AM–FM	Amplitude-modulated–frequency-modulated	
EDM	Electrical discharge machining	
EMD	Empirical mode decomposition	
FBG	Fiber Bragg grating	
LMD	Local mean decomposition	
MCKD	Maximum correlated kurtosis deconvolution	
MED	Minimum entropy deconvolution	
PF	Product function	
SNR	Signal-to-noise ratio	
VMD	Variational mode decomposition	
List of parameters		
**(1) Bearing geometry and fault-frequency parameters**		
Symbol	Definition	Unit
*B*	Bearing width	mm
*d*	Roller diameter	mm
*D_i_*	Diameter of the inner-ring raceway	mm
*D_o_*	Diameter of the outer-ring raceway	mm
*D_m_*	Bearing pitch diameter, $D_{m}\;=\;\frac{D_{i}+D_{o}}{2}$	mm
*Z*	Number of rolling elements	—
*α*	Bearing contact angle	rad
*f_i_*	Rotational frequency of the inner ring	Hz
*fo*	Rotational frequency of the outer ring	Hz
*V_i_*	Circumferential linear velocity of the inner-ring raceway	m/s
*V_o_*	Circumferential linear velocity of the outer-ring raceway	m/s
*V_c_*	Circumferential linear velocity of the bearing cage	m/s
*f_c_*	Cage rotational characteristic frequency	Hz
*f_bpfi_*	Inner-ring fault characteristic frequency	Hz
*f_bpfo_*	Outer-ring fault characteristic frequency	Hz
*f_bsf_*	Rolling-element fault characteristic frequency	Hz
**(2) FBG sensing parameters**		
Symbol	Definition	Unit
λ	Bragg wavelength	nm
Δλ	Bragg wavelength shift	nm
neff	Effective refractive index of the fiber core	—
Λ	Grating period	nm
α	Thermal expansion coefficient of the optical fiber	°C^−1^
ξ	Thermo-optic coefficient of the optical fiber	°C^−1^
Pe	Effective photo-elastic coefficient of the optical fiber	—
Δε	Axial strain variation	με
ΔT	Temperature variation	°C
**(3) LMD parameters**		
Symbol	Definition	Unit
*x*(*t*)	Original measured signal	με
*t*	Time	s
*n_i_*	Value of the *i*-th local extremum	με
*m_i_*	Local mean calculated from two adjacent extrema	με
*a_i_*	Local envelope value calculated from two adjacent extrema	με
*m*_11_(*t*)	Smoothed local mean function in the first decomposition process	με
*a*_11_(*t*)	Smoothed local envelope function in the first decomposition process	με
*s*_11_(*t*)	First demodulated signal	—
*a*_1*i*_(*t*)	Local envelope function obtained at the i-th iteration	με
*a*_1_(*t*)	Envelope signal of the first PF component	με
*s*_1*n*_(*t*)	Pure frequency-modulated signal obtained after n iterations	—
*PF*_1_(*t*)	First product function component	με
*PF_p_*(*t*)	The p-th product function component	με
*f*(*t*)	Instantaneous frequency of a PF component	Hz
*u*_1_(*t*)	Residual signal after extracting the first PF component	με
*u_k_*(*t*)	Final residual signal after extracting k PF components	με
**(4) PF selection and signal-reconstruction parameters**		
Symbol	Definition	Unit
*ρ*	Correlation coefficient between a PF component and the original signal	—
*ρi*	Normalized correlation coefficient of the i-th PF component	—
*RPFi,x*(*τ*)	Cross-correlation function between the i-th PF component and the original signal	με
*Rx,x*(*τ*)	Autocorrelation function of the original signal	με
*τ*	Time lag used in the correlation calculation	samples
*Ku_i_*	Raw kurtosis of the i-th PF component	—
$\tilde{{Ku}_{i}}$	Normalized kurtosis of the i-th PF component	—
*x_i_*	The i-th sample of the signal	με
*μ*	Mean value of the signal	με
*σ*	Standard deviation of the signal	με
*N_s_*	Number of samples used in the kurtosis calculation	—
*ω_i_*	Weighted index of the i-th PF component, $\omega_{i}=\tilde{{Ku}_{i}}\times \rho_{i}$	—
*ω_m_*	Mean value of the weighted indices	—
*N_PF_*	Number of PF components used in calculating *ω_m_*	—
**(5) MCKD parameters**		
Symbol	Definition	Unit
*CK_M_*(*T*)	Correlated kurtosis objective function	—
*MCKD_M_*(*T*)	Maximum correlated kurtosis obtained by optimizing the filter	—
*y_n_*	The nnn-th sample of the filtered or reconstructed signal	με
*N*	Total number of samples in the signal	—
*M*	Number of periodic offsets used in the correlated-kurtosis calculation	—
*T*	Deconvolution period expressed as the number of samples	samples
*T_s_*	Physical fault-impact period	s
*f_s_*	Sampling frequency	Hz
*f*	Finite impulse response filter coefficient vector	—
*f*	The l-th coefficient of the deconvolution filter	—
*L*	Length of the deconvolution filter	samples
*m*	Periodic-offset index in the MCKD objective function	—
*n*	Sample index	—
**(6) Quantitative evaluation parameters**		
Symbol	Definition	Unit
*A_bpfo_*	Spectral amplitude at the theoretical outer-ring fault characteristic frequency	Arbitrary amplitude unit
*R_f_*	Fault feature amplitude ratio, defined as the amplitude at fbpfo divided by the average amplitude of neighboring non-fault components	—
*SNR_f_*	Local signal-to-noise ratio corresponding to *R_f_*	dB
**Key experimental and algorithmic parameters**		
Parameter		Value used in this study
Test-bearing model		NJ205EM cylindrical roller bearing
Number of rollers, Z		13
Roller diameter, *d*		6.6 mm
Inner-ring raceway diameter, *Di*		31.5 mm
Outer-ring raceway diameter, *Do*		44.8 mm
Pitch diameter, *Dm*		38.15 mm
Bearing width, *B*		12 mm
Contact angle, *α*		0°
Shaft rotational speed		600 r/min
Shaft rotational frequency, *f_i_*		10 Hz
Sampling frequency, *f_s_*		8 kHz
Theoretical outer-ring fault frequency, *f_bpfo_*		Approximately 53.6 Hz
Artificial defect width		Approximately 1 mm
Artificial defect depth		Approximately 1 mm
Circumferential groove size		0.5 mm × 0.5 mm
FBG sensing length		Approximately 5 mm
Effective photo-elastic coefficient, *Pe*		Approximately 0.22
MCKD period, T		149 samples
MCKD filter length, L		100 samples
MCKD offset number, M		1
